# MiR-200/183 family-mediated module biomarker for gastric cancer progression: an AI-assisted bioinformatics method with experimental functional survey

**DOI:** 10.1186/s12967-023-04010-z

**Published:** 2023-03-02

**Authors:** Wenying Yan, Yuqi Chen, Guang Hu, Tongguo Shi, Xingyi Liu, Juntao Li, Linqing Sun, Fuliang Qian, Weichang Chen

**Affiliations:** 1 Department of Bioinformatics, School of Biology and Basic Medical Sciences, Suzhou Medical College of Soochow University, 199 Renai Road, Suzhou, 215123 China; 2 Center for Systems Biology, Soochow University, 199 Renai Road, Suzhou, 215123 China; 3 Department of Gastroenterology, The First Affiliated Hospital of Soochow University, 188 Shizi Road, Suzhou, 215006 China; 4 Jiangsu Institute of Clinical Immunology, The First Affiliated Hospital of Soochow University, Suzhou, 215021 China; 5 Suzhou Key Laboratory for Tumor Immunology of Digestive Tract, The First Affiliated Hospital of Soochow University, Suzhou, 215021 China; 6 Jiangsu Key Laboratory of Gastrointestinal Tumor Immunology, The First Affiliated Hospital of Soochow University, Suzhou, 215021 China; 7 Jiangsu Key Laboratory of Clinical Immunology, Soochow University, Suzhou, 215021 China; 8 Medical Center of Soochow University, Suzhou, 215000 China

**Keywords:** Gastric cancer, Network module, miR-200 and miR-183 families, CLLU1

## Abstract

**Background:**

Gastric cancer (GC) is a major cancer burden throughout the world with a high mortality rate. The performance of current predictive and prognostic factors is still limited. Integrated analysis is required for accurate cancer progression predictive biomarker and prognostic biomarkers that help to guide therapy.

**Methods:**

An AI-assisted bioinformatics method that combines transcriptomic data and microRNA regulations were used to identify a key miRNA-mediated network module in GC progression. To reveal the module’s function, we performed the gene expression analysis in 20 clinical samples by qRT-PCR, prognosis analysis by multi-variable Cox regression model, progression prediction by support vector machine, and in vitro studies to elaborate the roles in GC cells migration and invasion.

**Results:**

A robust microRNA regulated network module was identified to characterize GC progression, which consisted of seven miR-200/183 family members, five mRNAs and two long non-coding RNAs H19 and CLLU1. Their expression patterns and expression correlation patterns were consistent in public dataset and our cohort. Our findings suggest a two-fold biological potential of the module: GC patients with high-risk score exhibited a poor prognosis (*p-value* < 0.05) and the model achieved AUCs of 0.90 to predict GC progression in our cohort. In vitro cellular analyses shown that the module could influence the invasion and migration of GC cells.

**Conclusions:**

Our strategy which combines AI-assisted bioinformatics method with experimental and clinical validation suggested that the miR-200/183 family-mediated network module as a “pluripotent module”, which could be potential marker for GC progression.

**Supplementary Information:**

The online version contains supplementary material available at 10.1186/s12967-023-04010-z.

## Background

Gastric cancer (GC) remains a major global health problem and is the third leading cause of cancer-associated death worldwide [1]. Although recent advances in techniques have improved the prognosis of patients with GC, many patients are still diagnosed in advanced stages [2], and the mortality rate remains high because of the heterogeneity and complicated regulatory relations at the molecular level [3–6]. Thus, novel insights into the mechanisms underlying GC progression will be crucial.

Studies are increasingly characterizing the regulatory effects of non-coding RNAs in the initiation and development of GC, as well as drug resistance [7–12]. MicroRNAs (miRNAs) and long non-coding RNAs (lncRNAs) have received substantial attention. However, RNA molecules do not function in isolation and can be grouped into “competitive endogenous RNA networks” on the basis of the crosstalk between lncRNAs and mRNAs competing for shared miRNA response elements [13]. This lncRNA-miRNA-mRNA crosstalk, which is involved in various human cancers, may enable effective approaches to studying cancer pathogenesis and progression [14]. In GC, several of these regulatory axes have been determined to play roles in tumorigenesis and cancer progression; examples include LINC01234/miR-204-5p/CBFB [15], HOTAIR/miR-331-3p/HER2 [16], BC032469/miR-1207-5p/hTERT [17], and DLX6-AS1/miR-204-5p/OCT1 axis [18]. However, how lncRNA-miRNA-mRNA interactions control the regulatory mechanism of GC progression and the roles of these interactions have not been fully elucidated.

The bioinformatics methods that are with the help of miRNA-mediated regulated network (miRNet) enable study of the effects of RNA interactions in cancer at system level and global view, and may acid in the development of new therapeutic strategies and discovery of biomarkers. In past decades, several bioinformatics strategies have been proposed to identify module biomarkers or key modules for tumorigenesis and development, on the basis of miRNet. Cui et al. have integrated topological analysis and a random walk with restart algorithm to identify a prognostic signature for GC [19]. He et al. have identified a module using a clique-percolation method with CFinder software, to divide patients into groups according to survival outcomes [20]. Recently, Wang et al. have proposed the network-based matrix factorization framework NSOJNMF for miRNA-mediated regulated co-modules associated with the occurrence and development of cancer [21]. Most of the above methods take full advantage of network structures. Together with advances in “-omics” data, machine learning and AI techniques are powerful tools that can assess module biomarker discovery by integrating multimodal data.

**Fig. 1:**
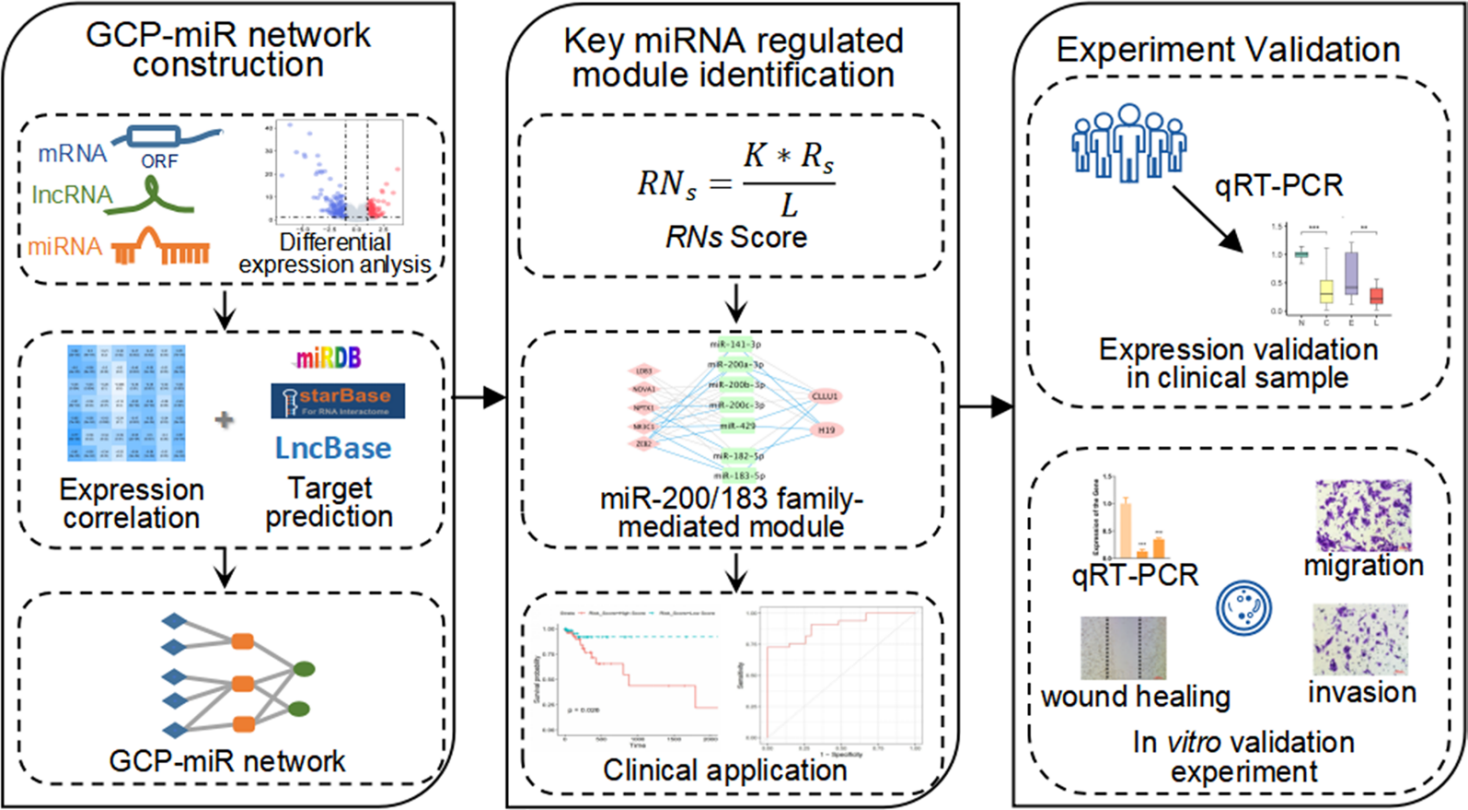
Overview of our method for identification of the key miRNA-mediated network module in GC progression

In this study, we identified a miRNet module to characterize GC progression by an AI-assisted bioinformatics method (Fig. 1) based on our previous designed scoring systems (*RNs*), which integrates various types of high-throughput data including transcriptomic, interactomic and network topological feature data [22]. Subsequently, we explored the prognostic and predictive roles of the module and validated the module in clinical samples and cell lines. Our findings suggested that miR-200/183 family-mediated network modules may have potential as biomarker for GC progression.

## Methods

### Derivation of the GC dataset

A data set containing the miRNA, mRNA and lncRNA expression profiles of 257 patients with TNM stage information from The Cancer Genome Atlas (TCGA) was used for identification of the miRNA-mediated network module [23]. The RNA counts were used for further analysis. The differentially expressed genes (DEGs) between early stage (stage I or II, 129 samples) GC groups (ESGC) and late stage (stage III or IV,128 samples) GC groups (LSGC) were identified with the R package DESeq2 [24], with the filter adjusted *p-value* < 0.05. The detail clinical information of the patients was listed in Additional file 1: Table S1. The association between gene expression and the clinicopathological features of GC patients was evaluated using the chi-square test.

### Construction and analysis of a GC progression-specific miRNA mediated (GCP-miR) network

A GCP-miR network was constructed in four steps: (1) the correlation between miRNAs and lncRNAs or mRNAs was determined with Spearman correlation tests, and pairs with *p-value* < 0.05 were retained and subjected to further steps. (2) The miRNA-mRNA interactions were selected by integrating the miRNA-mRNA pairs (Spearman’s correlation coefficient (*SCC*) < − 0.3) and the miRNA regulations predicted by miRDB (score > 50) [25, 26]. (3) To obtain more miRNA-lncRNA links, we retained the links meeting one of the following two criteria: (a) miRNA-lncRNA pairs with *SCC* less than − 0.3; and (b) miRNA-lncRNA pairs with *SCC* less than zero and were also predicted by starBase [27] or DIANA-LncBase [28]. (4) LncRNA-miRNA and mRNA-miRNA interactions that shared the same miRNAs were regarded as links in the GCP-miR network.

The R package igraph was used to calculate the topological parameter degree (*D*), betweenness (*B*) and closeness (*C*) for each node.

### Gene prioritization based on *RNs* score

The topological features from molecular networks alone are not sufficient to identify disease-associated genes without biological information. To overcome this limitation, we used *RNs* score (Eq. (1)) which integrated gene expression data via the SVM-RFE algorithm and topological characteristics of network nodes [22]. In our previous work, the *RNs* score was designed for protein–protein interaction networks. To further validate and extend the application of it, we applied the score to prioritize both coding and non-coding genes in the miRNA-mediated network.

1 $$RNs=\frac{K*{R}_{s}}{L}.$$

where *K* is the degree of a node in the network,* L* is the shortest path length of the node with the remaining nodes in the network, and *R*_*s*_ is the SVM-RFE score ranking the genes by expression level.

### Survival model construction

Using the selected genes fitted in a multivariable Cox regression model, we determined a risk score formula based on gene expression. Subsequently, each patient had a risk score, and the patients were divided into low-risk and high-risk groups according to a cutoff mean risk score. The Kaplan–Meier method was used to estimate the survival time and the log rank test was used to compare the survival difference between the low-risk and high-risk groups. A *p*-value < 0.05 was considered statistically significant.

### Functional enrichment analysis

Functional enrichment analysis and visualization were performed using R package clusterProfiler [29, 30]. Gene Ontology (GO) terms with adjusted *p-values* < 0.01 were considered significantly enriched, whereas Kyoto Encyclopedia of Genes and Genomes (KEGG) pathways with *p-values* < 0.05 were retained as significantly enriched pathways.

### Predictive model construction

To explore the predictive significance of gene combinations, we constructed an SVM predictive model on TCGA datasets and then evaluated its performance in our cohort with R package mlr3 [31]. The receiver operator characteristic (ROC) curve was plotted and the area under the curve (AUC) was calculated with R package ROCR [32].

### Sample collection and characterization

GC tissue samples and paired non-tumorous adjacent (NT) tissues (located 5 cm from the tumor margin) were obtained from patients with tissue pathology confirmation of GC at the First Affiliated Hospital of Soochow University (Suzhou, China) between March 2017 and August 2018. No patients had received radiotherapy or chemotherapy before surgery, and none of them had cardiac, liver or renal dysfunction. In the GC group, TNM-staging was determined according to the pathological staging criteria (version 8) of the American Joint Committee on Cancer. A total of 20 patients were finally enrolled in the analysis. The clinical characteristics of all patients were summarized in Additional file 1: Table S1.

### Cell culture and transfection

The cell lines GES-1, AGS, MKN-45, MKN-28 and HGC-27 were obtained from the American Type Culture Collection (Manassas, VA, USA). Cells were cultured in RPMI-1640 (Biological Industries, Beit Haemek, Israel) with 10% fetal bovine serum (Biological Industries) and 1% penicillin–streptomycin-amphotericin B (NCM Biotech, Suzhou, China, #C100C8) under 5% CO_2_ at 37 °C. Cells were transfected with CLLU1 siRNA, control siRNA (RiboBio, Guangzhou, China), has-miR-429 and has-miR-183-5p mimics or control mimics (Genepharma, Shanghai, China) with Lipofectamine 2000 (Invitrogen, Carlsbad, CA, USA) according to the manufacturer’s protocol. The sequences of mimics were provided in Additional file 1: Table S3.

### Transwell migration and invasion assays

Transwell migration and invasion assays were performed with Transwell plates (8.0 mm pore size, PET membrane, Falcon, USA). The lower chamber was filled with 400 µL RPMI 1640 containing 20% fetal bovine serum. Subsequently, 5 × 10^4^ cells in 400 µL serum-free medium were added to the upper chamber. After 24 h incubation at 37 °C, non-migrating cells were removed from the upper of membrane surface with a cotton swab. The filters were then fixed with 4% methanol for 15 min at room temperature, and stained with Crystal Violet for 10 min. Next, the membranes were washed with phosphate-buffered saline and allowed to dry, and an optical microscope (Olympus, Tokyo, Japan) was used to visualize the stained cells in five random fields on each membrane. Cells penetrating the membrane were counted at a magnification of 100× and the mean number was determined. For Transwell invasion assays, the membrane in the upper chamber was pre-coated with 50 µL Matrigel (Corning, Corning, NY, USA). All assays were performed in triplicate, and the experiment was repeated three times.

### Wound healing assay

Wound healing assays were performed to examine the migration ability of cells. Briefly, MKN-45 and HGC-27 cells were transfected with CLLU1 siRNA, or control siRNA for 48 h, then seeded in 12-well plates. When the cells reached 90–95% confluence, a single scratch wound was made across the plate surface with a 200-μL pipette tip. The scratch wounds were photographed over a 48-h period using an inverted microscope (Olympus), and the wound width of was quantified with imaging software. Each assay was performed in triplicate.

### RNA extraction and qRT-PCR analysis

Total RNA was extracted from tissue samples with TRIzol reagent (TaKaRa) according to the manufacturer’s instructions. For mRNA and lncRNA expression, 1 μg total RNA was reversed transcribed into cDNA with PrimeScript RT Master Mix (Takara). The qRT-PCR was performed in a CFX96 TouchTM real-time PCR system (Bio-Rad, Hercules, CA, USA) with SYBR Green Master Mix (Vazyme, Nanjing, China). For miRNA expression analysis, 1 µg total RNA was used for first-strand DNA synthesis with a miRNA 1st Strand cDNA Synthesis Kit (Vazyme), and qRT-PCR was performed with miRNA universal SYBR qPCR Master Mix (Vazyme). Relative gene expression was calculated using the 2^−ΔΔCt^ method, with β-actin and small nuclear RNA U6 used as endogenous controls for mRNA/lncRNA and miRNA. The primer sequences for qRT-PCR are provided in Additional file 1: Table S2. The Wilcoxon rank sum test was used to test the difference between the GC and NT groups, as well as the ESGC and LSGC groups.

## Results

### GCP-miR network construction

Expression data for lncRNAs, mRNAs and miRNAs were collected from 129 patients with ESGC and 128 patients with LSGC in TCGA. First, the significant DEGs between ESGC and LSGC were identified. A total of 1165 mRNAs (649 up-regulated and 516 down-regulated), 15 lncRNAs (11 up-regulated and four down-regulated) and 59 miRNAs (33 up-regulated and 26 down-regulated) were found to be differentially expressed between two groups and the miRNA-lncRNA and miRNA-mRNA pairs with significant negative correlations were used in subsequent analyses.

**Fig. 2:**
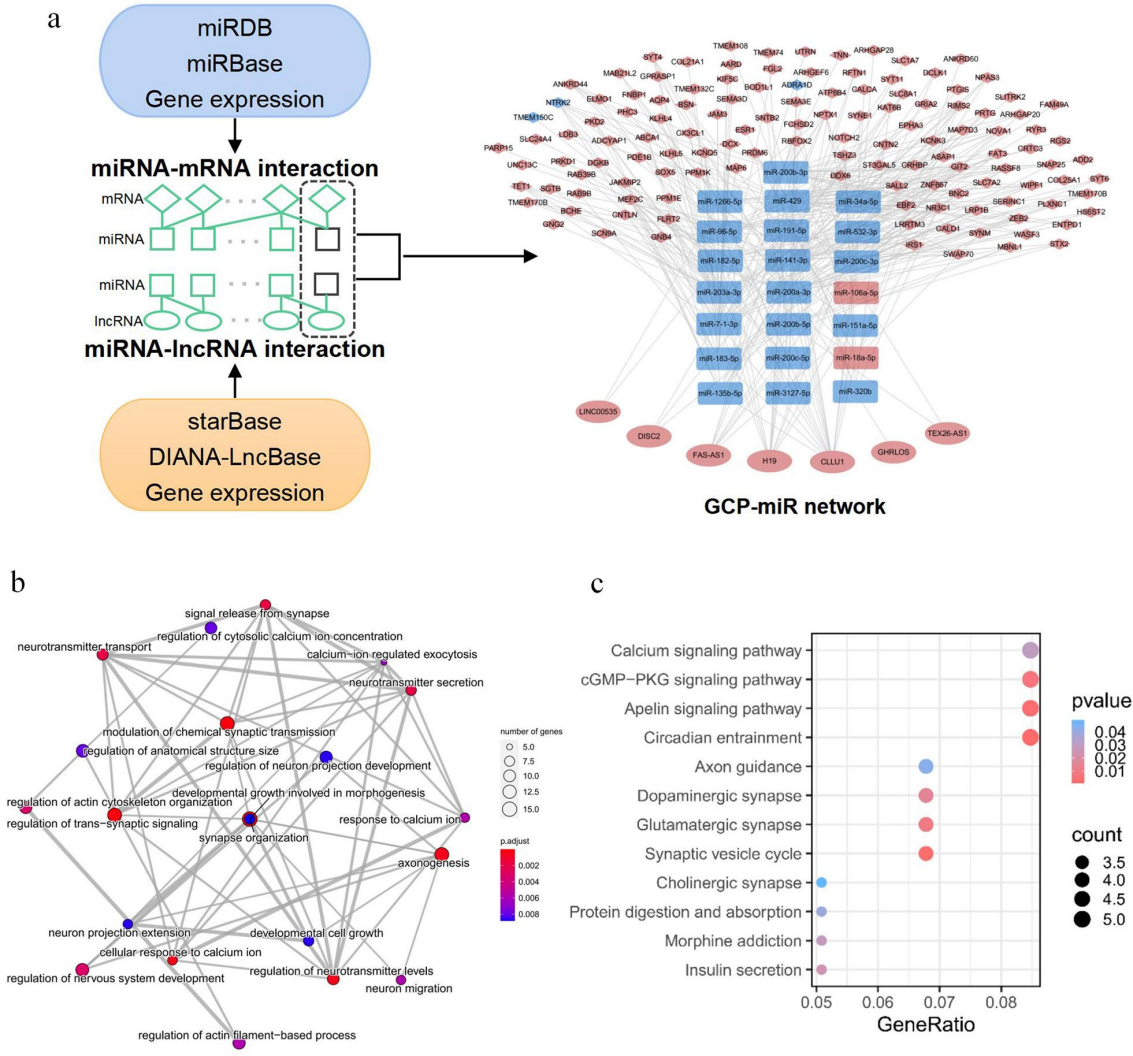
GCP-miR network and its biological function. **a** Construction and visualization of the GCP-miR network. Diamonds, rectangles, and ellipses indicate mRNAs, miRNAs and lncRNAs, respectively. Pink represents high expression, and blue represents low expression in LSGC compared with ESGC. **b** GO biological process enrichment analysis of genes in the GCP-miR network (adjusted *p-value* < 0.01). Each node represents a GO term and each edge represents the overlap between two terms. **c** KEGG-based enrichment analysis of genes in the GCP-miR network (*p-value* < 0.05). KEGG terms were sorted by gene ratio

We then established the GCP-miR network by integrating the above pairs and the results from miRNA target prediction tools as described in Methods. The final GCP-miR network consisted of three types of nodes (22 miRNAs, 126 mRNAs, and 7 lncRNAs), and two type of links (295 miRNA-mRNA and 46 miRNA-lncRNA links; Fig. 2a).

Functional enrichment analysis was performed on genes in the GCP-miR network to explore their biological functions. As shown in Fig. 2b, c, the GO biological process terms and KEGG pathways were highly enriched in cancer development and progression associated pathways, such as circadian entrainment [33], the apelin signaling pathway [34, 35], and the cGMP-PKG signaling pathway [36]. Notably, beyond the cancer-associated pathways, several nervous system-associated terms were enriched, such as glutamatergic synapse. These results indicated that the GCP-miR network is involved in GC progression.

### The miR-200/183 family miRNAs are key in GC progression

**Fig. 3:**
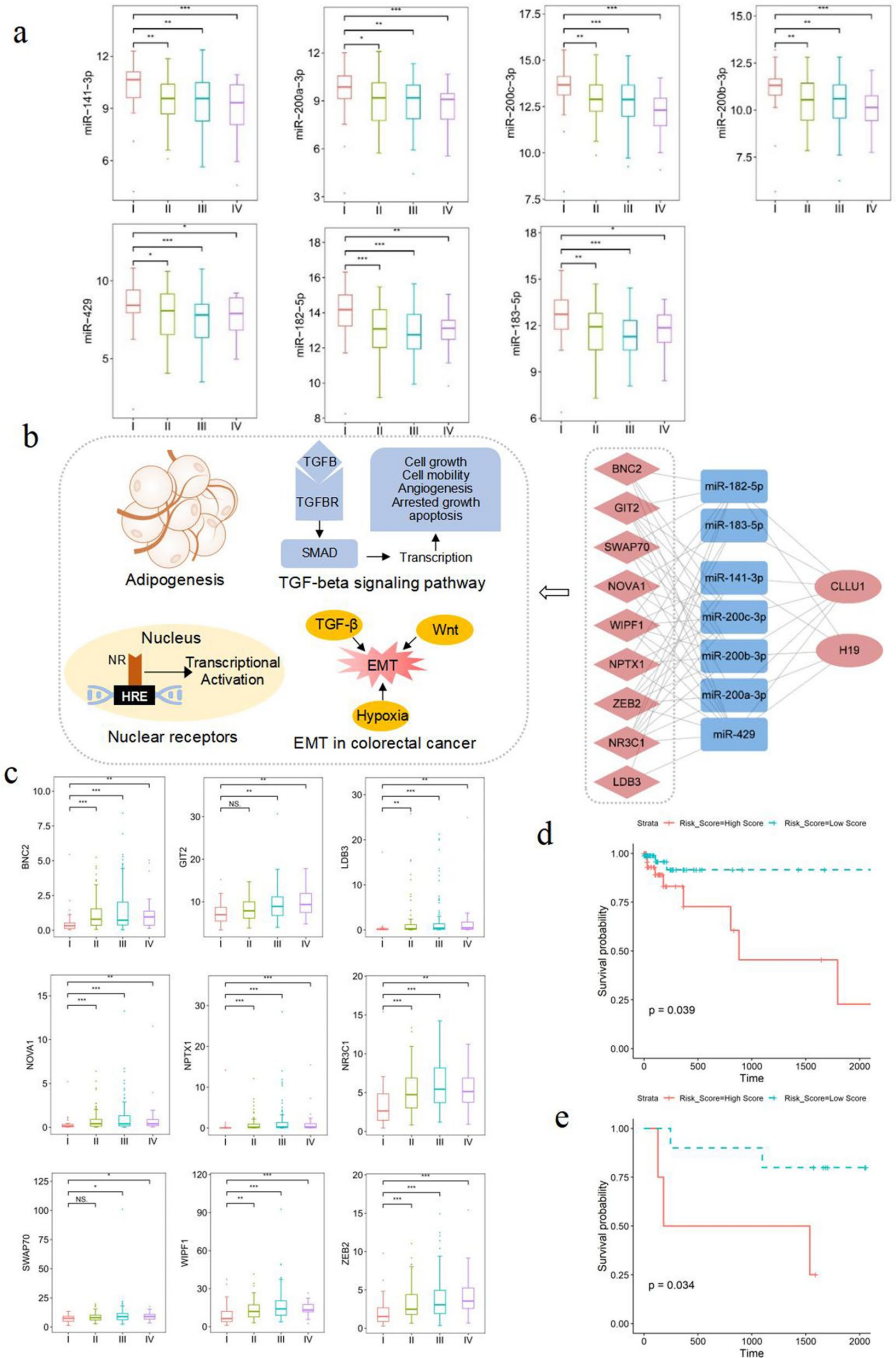
The miR-200/183 family-mediated module is key module in GC progression. **a** Expression of miR-200 and miR-183 families in GC tissues with four TNM stages. **b** The miR-200/183 family-mediated module for GC progression. Pink and blue denote up and down regulation, respectively, in LSGC samples. **c** Expression of nine mRNAs in GC tissues with four TNM stages. **d** Kaplan–Meier analysis was used to estimate the survival of high-risk vs. low-risk patients with GC according to the seven miRNA signatures from the miR-200 and miR-183 families in the training set. **e** Kaplan–Meier analysis was used to estimate the survival of high-risk vs. low-risk patients with GC according to the seven miRNA signatures from the miR-200 and miR-183 families in the validation cohort. (*p-value < 0.05, **p-value < 0.01, ***p-value < 0.001)

To identify key genes associated with GC progression, we calculated the *RNs* score for each gene in the GCP-miR network, which fully accounts for the network topological structure and gene expression levels in cancer samples. The top 10 genes with highly *RNs* in the network are listed in Table 1, including eight down-regulated miRNAs and two up-regulated lncRNAs in LSGC samples. Because miR-203a-3p had the highest *RNs* score, and has been reported to predict metastases and poor prognosis in human GC clinical samples [37]. We selected the remaining nine genes for further analysis. Notably, the remaining seven miRNAs were grouped into two families: miR-200 and miR-183 (Table 1) All seven miRNAs were significantly up-regulated in TNM stage I than in other stages (Fig. 3a). Previous studies have demonstrated that down-regulation of miR-200 family members promotes GC progression in *vitro* in GC cell lines [38–41] and characterize sub-types of GC with poor-prognosis [42]. Moreover, miR-182-5p and miR-183-5p are involved in GC cell proliferation and have been significantly negatively correlated with ETM scores in lung cancer [43], but their expression patterns have not been consistent in across prior studies [44–46]. Beyond miRNAs, two lncRNAs H19 and CLLU1 had high *RNs* score. H19 affects GC cell proliferation and contributes to GC progression [47, 48]. Although no evidence has indicated a role of CLLU1 in GC progression, it has been reported to be associated with hepatocellular carcinoma prognosis [49]. Its roles in GC cells will are explored below.

**Table 1:** Top ten genes ranked by *RNs* score

Name	*D*	*B*	*C*	*RNs*	Family	Function in GC cell
miR-203a-3p	25	0.175	0.394	9.847	miR-203	Proliferation, cycle and apoptosis [57]
miR-200c-3p	24	0.063	0.386	8.421	miR-200	Growth and invasion [39]
miR-200a-3p	25	0.083	0.394	7.609	miR-200	Proliferation, cell cycle and migration [40]
miR-141-3p	28	0.110	0.400	7.127	miR-200	Proliferation, invasion, migration and metastasis [38]
miR-200b-3p	23	0.058	0.390	6.522	miR-200	Growth and invasion [39]
miR-182-5p	17	0.083	0.378	6.140	miR-183	Proliferation and colony formation [45]
miR-429	28	0.098	0.400	5.091	miR-200	Proliferation and viability [41]
miR-183-5p	15	0.063	0.361	4.426	miR-183	Proliferation and migration [46]
H19	13	0.145	0.428	5.561	lncRNA	Proliferation and apoptosis [47, 58]
CLLU1	17	0.195	0.453	5.500	lncRNA	–

### The miR-200/183 family-mediated module is key in GC progression

Because of the important roles of the seven miRNAs and two lncRNAs with high *RNs* score in GC progression, we selected them and mRNAs, which were interacted with members from both miR-200 and miR-183 families in GCP-miR network, as the key module for GC progression. As shown in Fig. 3b, nine mRNAs were added in the module, which were also significantly up-regulated in LSGC (Fig. 3c) and involved in the adipogenesis, TGF-beta signaling, nuclear receptor and EMT in colorectal cancer pathways. The results indicate that miR-200/183 family-mediated module might contribute the GC progression by regulating their target genes. Moreover, in the module, the gene expression showed significantly negative correlations of lncRNAs and mRNAs with miR-200/183 family members, and significantly positive correlations between miRNAs in GC samples (Additional file 1: Fig. S2).

Then we examined the relationship between miR-200/183 family-mediated module’s members and clinical characteristics of the patients in TCGA dataset. The results (Table 2) showed that the expression level of all the miRNAs were significantly correlated with the age of patients (p < 0.05), six of the seven miRNAs were significantly correlated with the TNM stage (p < 0.05), and five miRNAs were significantly correlated with histological grade. For lncRNAs, CLLU1 expression levels were correlated with histological type (p = 0.041) and grade (p = 0.044). Moreover, the TNM stages and histological grade had also significantly relation with most of the mRNAs in the module (Table 3).

**Table 2:** Association of miRNAs and lncRNAs in miR-200/183 family-mediated module with clinic pathological characteristics of GC patients from TCGA

Characteristics	Total	miR-182-5p			miR-183-5p			miR-200b-3p			miR-141-3p			miR-200c-3p			miR-200a-3p			miR-429			CLLU1			H19		
		High	Low	p	High	Low	p	High	Low	p	High	Low	p	High	Low	p	High	Low	p	High	Low	p	High	Low	p	High	Low	p
Age																												
≤ 60	89	36	53	0.004*	37	52	0.009*	39	50	0.006*	42	47	0.041*	39	50	0.011*	39	50	0.002*	42	47	0.033*	22	67	0.433	14	75	0.266
> 60	168	101	67		100	68		105	63		103	65		103	65		109	59		104	64		33	135		17	151	
Gender																												
Female	99	53	46	1.000	54	45	0.852	56	43	0.994	56	43	1.000	51	48	0.409	59	40	0.699	59	40	0.559	24	75	0.470	13	86	0.826
Male	158	84	74		83	75		88	70		89	69		91	67		89	69		87	71		31	127		18	140	
TNM stage																												
I	35	28	7	0.003*	27	8	0.007*	28	7	0.006*	27	8	0.039*	29	6	0.001*	26	9	0.193	28	7	0.028*	4	31	0.369	2	33	0.193
II	94	51	43		52	42		50	44		53	41		50	44		52	42		51	43		19	75		8	86	
III	103	45	58		45	58		57	46		54	49		55	48		57	46		53	50		26	77		17	86	
IV	25	13	12		13	12		9	16		11	14		8	17		13	12		14	11		6	19		4	21	
T																												
T1	10	10	0	0.001*	10	0	0.003*	10	0	0.002*	10	0	0.003*	10	0	0.002*	9	1	0.053	9	1	0.006*	0	10	0.290	0	10	0.068
T2	64	42	22		40	24		43	21		43	21		42	22		42	22		45	19		13	51		3	61	
T3	106	53	53		53	53		49	57		56	50		56	50		55	51		54	52		22	84		18	88	
T4	77	32	45		34	43		42	35		36	41		34	43		42	35		38	39		20	57		10	67	
N																												
N0	87	54	33	0.249	51	36	0.655	56	31	0.208	52	35	0.256	56	31	0.177	54	33	0.793	52	35	0.874	17	70	0.664	8	79	0.692
N1	75	36	39		39	36		37	38		45	30		40	35		41	34		43	32		15	60		9	66	
N2	44	23	21		23	21		22	22		26	18		23	21		25	19		23	21		9	35		7	37	
N3	50	24	26		24	26		29	21		22	28		23	27		28	22		28	22		14	36		7	43	
M																												
M0	230	126	104	0.195	125	105	0.207	130	100	0.533	133	97	0.324	131	99	0.255	133	97	0.971	131	99	0.246	50	180	0.568	27	203	0.674
M1	16	8	8		9	7		7	9		8	8		6	10		9	7		11	5		4	12		3	13	
Mx	11	3	8		3	8		7	4		4	7		5	6		6	5		4	7		1	10		1	10	
Type																												
Diffuse	49	21	28	0.212	19	30	0.023*	23	26	0.178	24	25	0.108	22	27	0.076	23	26	0.149	23	26	0.059	17	32	0.041*	4	45	0.636
Intestinal	89	52	37		56	33		56	33		58	31		57	32		57	32		59	30		16	73		12	77	
Others	119	64	55		62	57		65	54		63	56		63	56		68	51		64	55		22	97		15	104	
Grade																												
G1	3	0	3	0.056	1	2	0.029*	0	3	0.029*	1	2	0.034*	0	3	0.000*	0	3	0.030*	2	1	0.226	0	3	0.044*	1	2	0.631
G2	79	48	31		52	27		53	26		55	24		58	21		54	25		52	27		9	70		10	69	
G3	170	88	82		83	87		88	82		87	83		81	89		91	79		90	80		45	125		19	151	
Gx	5	1	4		1	4		3	2		2	3		3	2		3	2		2	3		1	4		1	4	

T: Primary tumor invasion depth; N: Lymph node metastasis; M: Distant metastasis

^*^p-value < 0.05

**Table 3:** Association of mRNAs in miR-200/183 family-mediated module with clinic pathological characteristics of GC patients from TCGA

Characteristics	Total	BNC2			GIT2			LDB3			NOVA1			NPTX1			NR3C1			SWAP70			WIPF1			ZEB2		
		High	Low	p	High	Low	p	High	Low	p	High	Low	p	High	Low	p	High	Low	p	High	Low	p	High	Low	p	High	Low	p
Age																												
≤ 60	89	37	52	0.003*	51	38	0.004*	22	67	0.076	33	56	0.005*	31	58	0.000*	48	41	0.034*	35	54	0.964	50	39	0.003*	42	47	0.119
> 60	168	39	129		63	105		25	143		34	134		24	144		66	102		68	100		61	107		61	107	
Gender																												
Female	99	29	70	1.000	46	53	0.682	20	79	0.644	28	71	0.622	23	76	0.681	44	55	1.000	42	57	0.633	45	54	0.652	42	57	0.633
Male	158	47	111		68	90		27	131		39	119		32	126		70	88		61	97		66	92		61	97	
TNM stage																												
I	35	3	32	0.025*	9	26	0.023*	1	34	0.047*	3	32	0.042*	2	33	0.084	6	29	0.003*	9	26	0.071	8	27	0.050	7	28	0.023*
II	94	28	66		38	56		16	78		24	70		20	74		41	53		33	61		40	54		35	59	
III	103	36	67		52	51		24	79		34	69		27	76		55	48		49	54		51	52		48	55	
IV	25	9	16		15	10		6	19		6	19		6	19		12	13		12	13		12	13		13	12	
T																												
T1	10	0	10	0.011*	2	8	0.002*	0	10	0.008*	0	10	0.044	0	10	0.028*	1	9	0.005*	3	7	0.028*	1	9	0.004*	2	8	0.005*
T2	64	13	51		21	43		4	60		11	53		8	56		20	44		16	48		21	43		19	45	
T3	106	32	74		44	62		24	82		32	74		24	82		52	54		49	57		45	61		39	67	
T4	77	31	46		47	30		19	58		24	53		23	54		41	36		35	42		44	33		43	34	
N																												
N0	87	19	68	0.101	32	55	0.329	12	75	0.200	16	71	0.079	17	70	0.965	25	62	0.005*	27	60	0.150	35	52	0.853	29	58	0.196
N1	75	22	53		34	41		12	63		21	54		17	58		39	36		33	42		32	43		29	46	
N2	44	13	31		23	21		8	36		10	34		9	35		22	22		22	22		19	25		18	26	
N3	50	21	29		24	26		14	36		19	31		11	39		27	23		21	29		24	26		26	24	
M																												
M0	230	68	162	0.558	102	128	0.075	44	186	0.276	61	169	0.823	52	178	0.196	101	129	0.543	88	142	0.150	101	129	0.206	92	138	0.140
M1	16	6	10		10	6		3	13		4	12		3	13		9	7		10	6		8	8		9	7	
Mx	11	2	9		2	9		0	11		2	9		0	11		4	7		5	6		2	9		2	9	
Type																												
Diffuse	49	28	21	0.000*	32	17	0.002*	14	35	0.087	24	25	0.000*	15	34	0.167	30	19	0.007*	26	23	0.114	30	19	0.003*	32	17	0.000*
Intestinal	89	13	76		30	59		12	77		12	77		15	74		30	59		34	55		28	61		25	64	
Others	119	35	84		52	67		21	98		31	88		25	94		54	65		43	76		53	66		46	73	
Grade																												
G1	3	1	2	0.000*	1	2	0.000*	1	2	0.000*	1	2	0.001*	2	1	0.000*	1	2	0.001*	1	2	0.061	1	2	0.001*	1	2	0.000*
G2	79	7	72		16	63		3	76		8	71		5	74		20	59		22	57		19	60		13	66	
G3	170	65	105		94	76		40	130		55	115		46	124		90	80		78	92		88	82		86	84	
Gx	5	3	2		3	2		3	2		3	2		2	3		3	2		2	3		3	2		3	2	

T: primary tumor invasion depth; N: lymph node metastasis; M: distant metastasis

^*^p-value < 0.05

Finally, we investigate the prognostic values of the module in the TCGA dataset and our validation cohort. For TCGA datasets, patients were randomly allocated to the training (n = 180) or testing (n = 77) cohorts using a 7:3 ratio. A risk score formula based on the expression level of miRNAs in the training cohort was created as follows by multi-variable Cox regression model: Risk score = (0.0316 × miR-200b-3p) + (0.0716 × miR-141-3p) + (− 0.0193 × miR-200c-3p) + (− 0.1105 × miR-200a-3p) + (0.1529 × miR-429) + (− 0.5501 × miR-182-5p) + (0.3274 × miR-183-5p). The HR and 95% confidence interval for each miRNA were listed in Additional file 1: Table S4. Then the score for each patient was calculated, and the patients were assigned to high-risk score or low-risk score groups according to the median value of risk score (− 2.4568) in the training cohort. The Kaplan–Meier curves showed the high-risk group had significantly shorter overall survival than the low-risk group in the training group (p-value = 0.039; Fig. 3d), the testing group (p-value = 0.042; Additional file 1: Fig. S3a), and the whole group (p-value = 0.005; Additional file 1: Fig. S3b). For our validation cohort, the results were consistent with those of the TCGA, that is, low-risk score groups exhibited better survival than the high-risk groups (p-value = 0.034; Fig. 3e).

### Validation of the miR-200/183 family-mediated module in GC progression

**Fig. 4:**
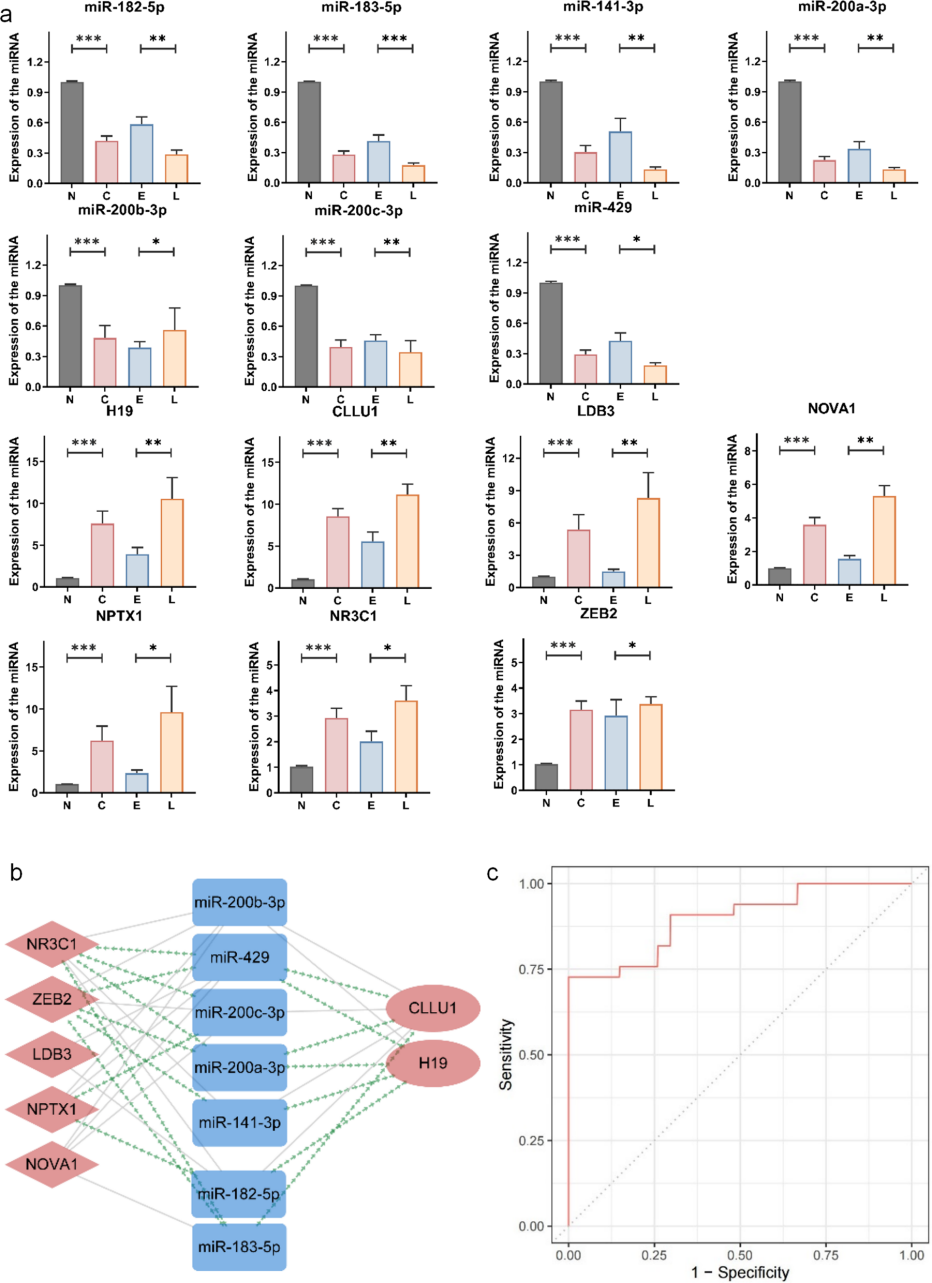
Validation of the miR-200/183 family-mediated module in clinical GC samples. **a** Expression levels of genes in the miR-200/183 family-mediated module (*p-value < 0.05, **p-value < 0.01, ***p-value < 0.001). N: NT samples; C: GC samples; E: ESGC samples; L: LSGC samples. **b** Validation of the miR-200/183 family-mediated module in GC progression. Green links denote significantly negative correlations with *p-values* less than 0.05 and SCC less than -0.5. **c** ROC curve of six gene signatures to stratify the ESGC and LSGC samples

To ascertain the role of the miR-200/183 family-mediated module in human GC progression, we further validated the module by using newly collected samples from patients with GC. The expression levels of seven miRNAs, the two lncRNAs H19 and CLLU1, and five randomly selected mRNAs (LDB3, NOVA1, NPTX1, NR3C1 and ZEB2) in 20 pairs of GC and NT tissues were measured. The miR-200 and miR-183 families members were significantly lower in cancer tissues than NT tissues, and also were significantly lower in LSGC than ESGC. In contrast, their potential targets, two lncRNAs and five mRNAs, were significantly up-regulated in the GC and LSGC with respect to NT tissues and ESGC, respectively (Fig. 4a, *p-value* < 0.05), thus highlighting the specificity of these candidate biomarkers for GC progression.

We then performed correlation analysis to validate the association among the genes in the module in our cohort (Additional file 1: Fig. S4). The interactions between the miRNAs and their targets with *p-value* < 0.5 and SCC < − 0.5 were marked as green lines in the module (Fig. 4b). In agreement with the results based on TCGA datasets, lncRNAs H19 and CLLU1 showed a significant negative correlation with the miR-200 and miR-183 families. Similarly, most mRNAs and miRNAs also showed negative correlations in our cohort. The miR-200 family members displayed significant positive correlations with miR-183 family.

Finally, to evaluate the predictive ability of the module in the classification of ESGC and LSGC, we constructed SVM models according to the expression levels of genes in the module. We first constructed the predictive models with SVM for all combinations of 14 genes in the module with the dataset from TCGA, then evaluated the predictive ability of the combinations to stratify ESGC and LSGC in our independently collected GC samples. Finally, the combination of six genes miR-182-5p, miR183-5p, LDB3, NOVA1, NPTX1 and NR3C1 achieved the highest AUC (0.90, Fig. 4c) among all combinations, thus indicating their ability to predict GC progression.

### The miR-200/183 family-mediated module influence the invasion and migration of GC cells

**Fig.5:**
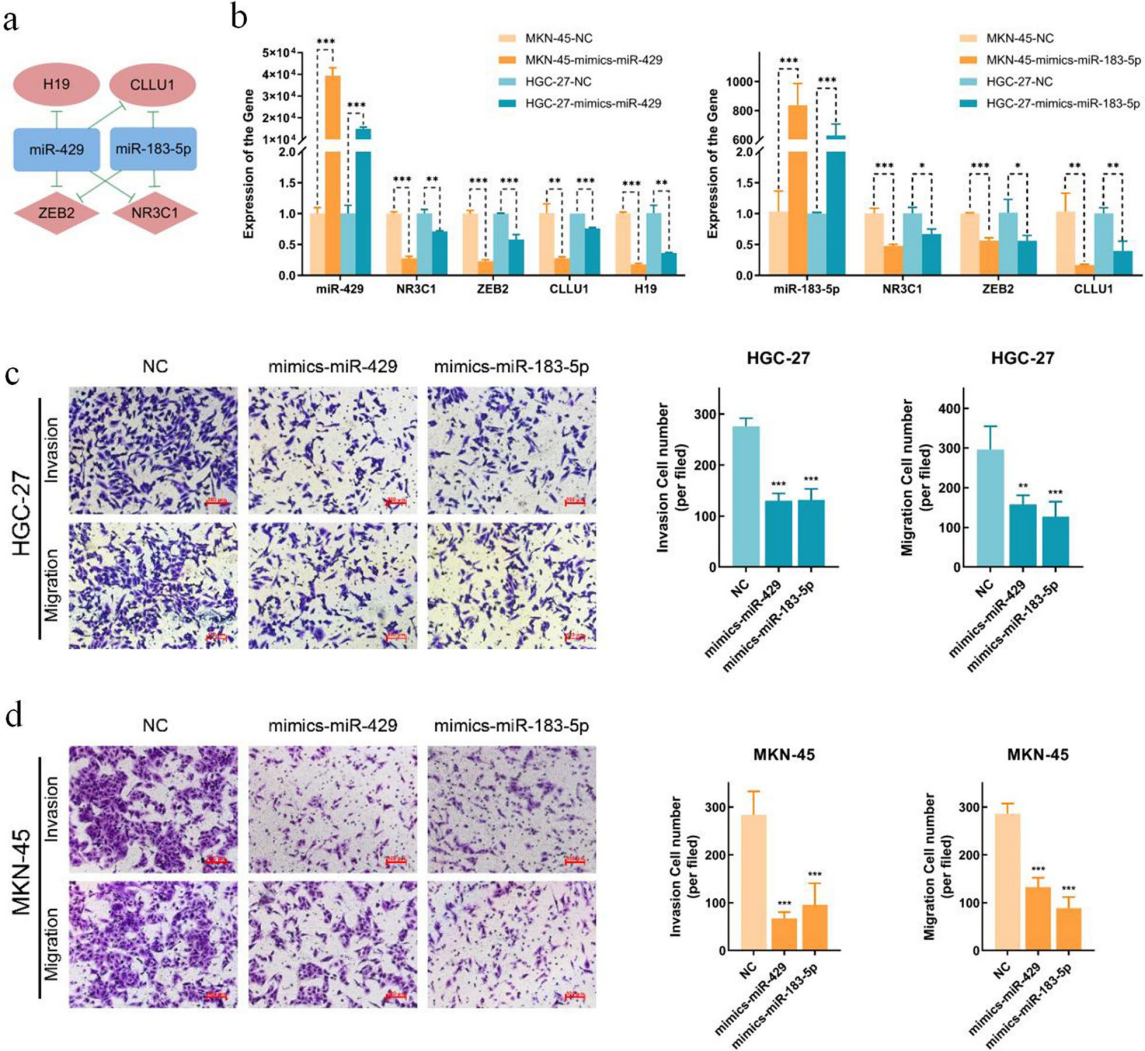
Evaluation the effects of miR-200/183 family-mediated module effects on invasion and migration of GC cells. **a** miR-429 and miR-183-5p regulated sub-module. **b** Expression of miR-429, miR-183-5p and their targets in miR-429 or miR-183-5p mimics-transfected MKN-45 cells and HGC-27 cells. **c**, **d** Transwell migration and invasion assays showed that miR-429 mimics and miR-183-5p inhibited the migratory and invasive capacity of MKN-45 and HGC-27 cells. The data represent means ± SD. *p-value < 0.05; **p-value < 0.01; ***p-value < 0.001

To further validate the biological functions of the miR-200/183 family-mediated module in GC, we performed functional analysis for the RNAs that involved in it. As shown in Table 1, most of the miRNAs in the module, such as miR-200a-3p and miR-141-30, have been reported to affects the invasion and migration of GC cells in multiple studies. Therefore, we selected miR-429 and miR-183-5p as representatives of the miR-200 and miR-183 families and explore their function in GC cells, as well as their predicted targets as shown in Fig. 5a. MKN-45 and HGC-27 cells were transfected with miR-429 mimics, miR-183-5p mimics, or control mimics (Fig. 5b). Transwell invasion and migration assays were then performed to examine the migratory and invasive ability in vitro. As shown in Fig. 5c, d, the invasion and migration of GC cells was more suppressed in the miR-429 and miR-183-5p mimics groups than in the controls (*p-*value < 0.01). These results indicated that the expression of miR-429 and miR-183-5p efficiently weakened the metastatic potential of GC cells.

We next validated the interactions among the miRNAs and their targets in Fig. 5a by evaluating the expression of the targets in GC cells that were transfected with miR-429 mimics and or miR-183-5p mimics. We observed that both miRNA mimics significantly decreased the expression of their targets (Fig. 5b). The expression levels of miR-429 targets, including NR3C1, ZEB2, CLLU1 and H19, were significantly lower than those in the NC groups. Similarly, the expression of miR-183-5p targets NR3C1, ZEB2 and CLLU1 in the corresponding mimic group was also significantly lower than that in the NC groups. These expression patterns confirmed the potential interactions among the miRNAs and their targets in GC cells.

**Fig. 6:**
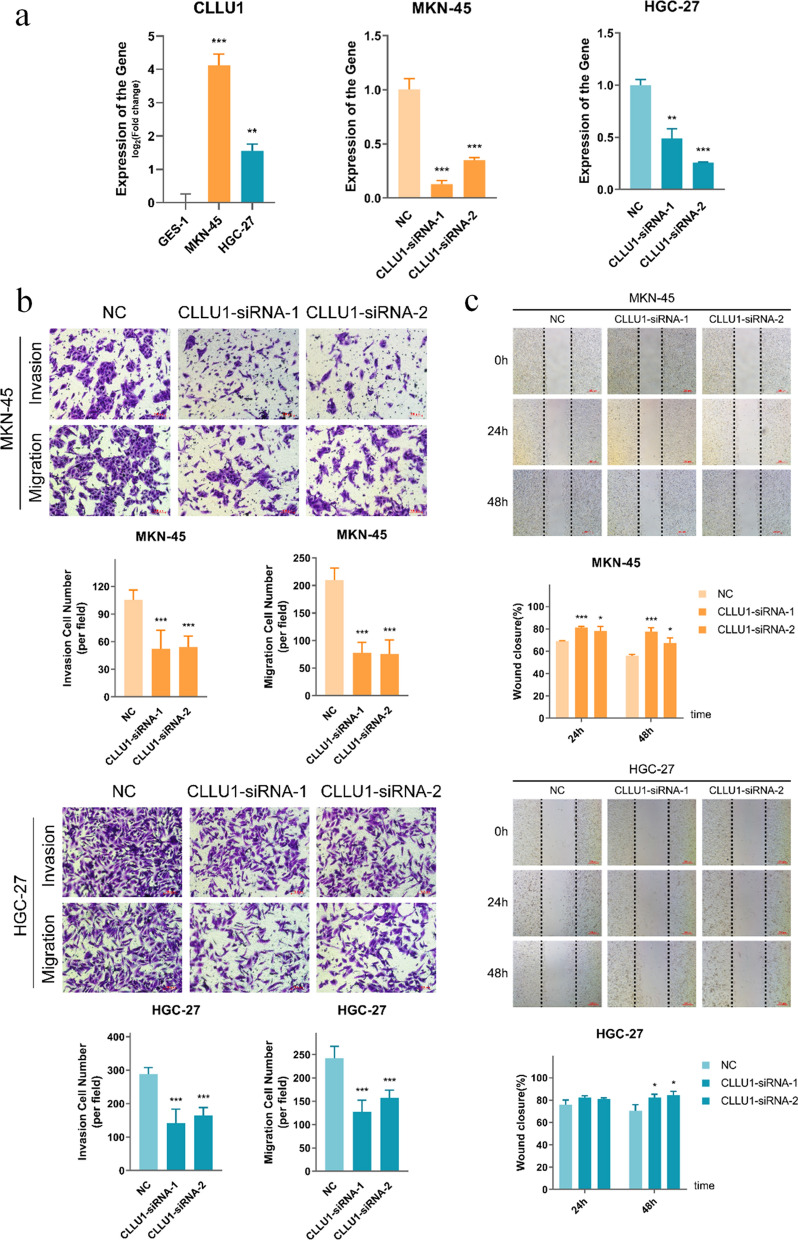
Knockdown of CLLU1 inhibits the invasion and migration of GC cell lines. **a** Expression of CLLU1 in GC cell lines and its expression after knockdown by siRNA. **b** Transwell migration and invasion assays indicating that knockdown of CLLU1 inhibits the migratory and invasive ability of GC cell lines. **c** Wound healing assay indicating that knockdown of CLLU1 impairs the migratory ability of GC cell lines. The data represent means ± SD. *p-value < 0.05; **p-value < 0.01; ***p-value < 0.001

Finally, we explored the functions of targets of miR-429 and miR-183-5p in GC cells. Because several studies have reported that high expression of their targets such as ZEB2 [50, 51] and H19 [48, 52] could promote the GC progression, we performed functional analysis of another lncRNA CLLU1 in the module. CLLU1 showed elevated expression in MKN-45 and HGC-27 cell lines compared with normal gastric cells (Fig. 6a). The expression levels of eight other genes with high *RNs* scores were also measured in GC cell lines (Additional file 1: Fig. S1). We transfected siRNAs to knock down CLLU1 in MKN-45 and HGC-27 (Fig. 6a). Transwell and wound healing assays were then performed to examine the in vitro migratory and invasive ability of CLLU1. As shown in Fig. 6b, c, the invasion and migration of GC cells was greatly suppressed in the knock down group (*p-value* < 0.01). These results indicated that inhibition of CLLU1 efficiently weakened the metastatic potential of GC cells. Taken together, these results reveal that the miR-429 and miR-183-5p regulated sub-module contribute to the invasion and migration of GC cells.

## Discussion

GC is a major cause of global mortality and remains a major health burden in Asian countries including China. It is often diagnosed in advanced stages. Because molecular events in GC progression are promoted by complex genomic interactions, molecules can be grouped into “networks” according to their interactions that contribute to cancer progression; several network-based computational methods have been proposed [53]. The miRNAs interact with different molecules and produce varying outcomes depending on the tumor microenvironment [54–56]. In this study, we attempted to delineate the miRNA-mediated molecular mechanism operating in GC progression with an AI-assisted bioinformatics method to integrate transcriptomic, interactomic and network topological feature data. Some major findings are listed as follows.

We first identified the key genes in GC progression by using our previously designed *RNs* score, which considered both the topological characteristics of genes in the GCP-miR network and the expression profiles of genes in GC samples. Seven miRNAs from the miR-200 and miR-183 families had high *RNs* scores, thus indicating their important topological roles in the network. These seven miRNAs showed significantly different expression levels during GC progression. Indeed, we validated their significant down-regulation in LSGC in our cohort, and observed robust correlations among them. The results confirmed their important roles in GC progression, thus providing a molecular network perspective corroborating findings from previous reports [38–41, 44–46].

**Fig. 7:**
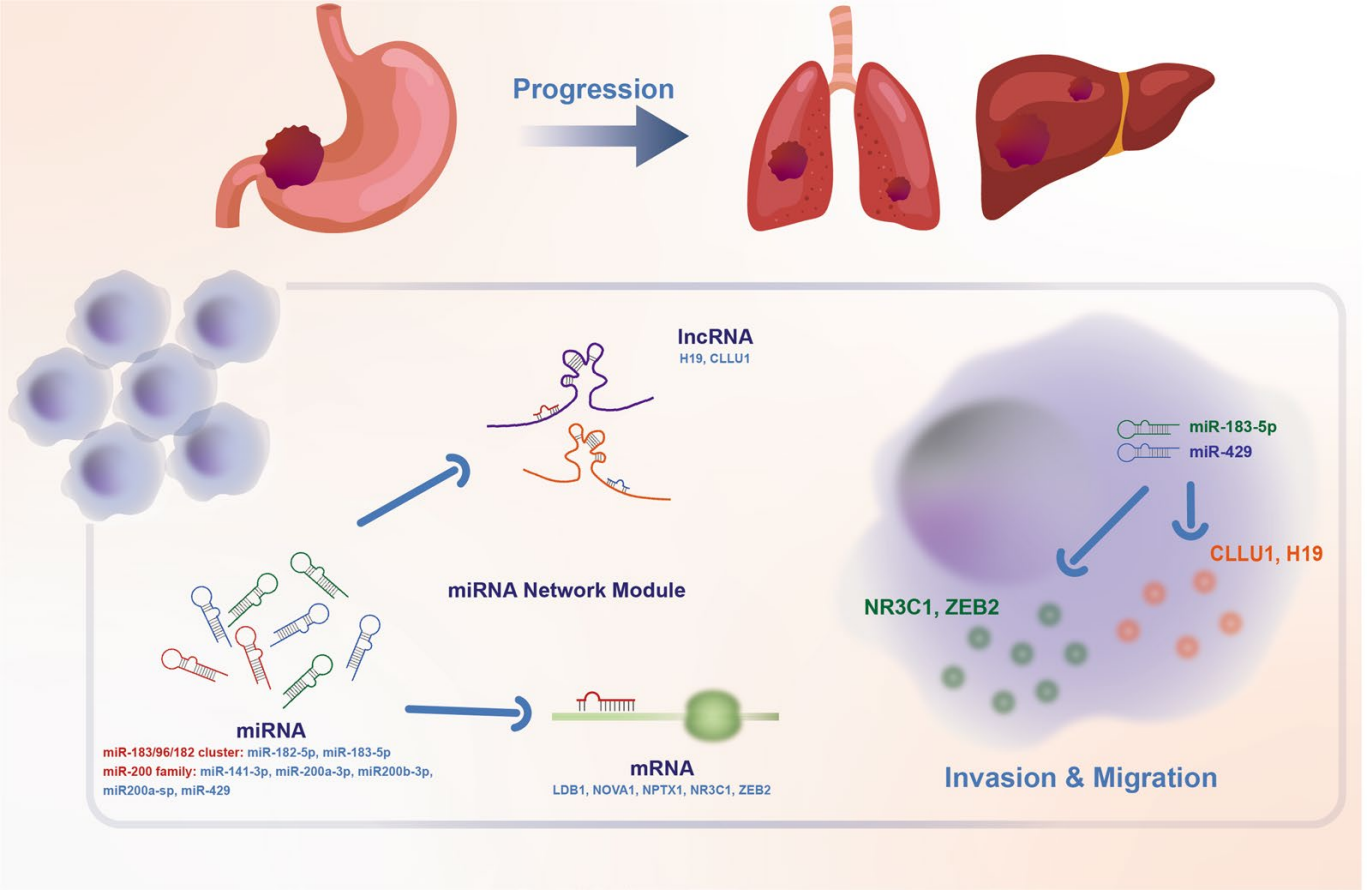
Schematic diagram of miR-200/183 family-mediated module promoting GC progression

Subsequently, we identified the module for GC progression by selecting the identified miRNAs and their target genes, both of which were regulated by members of the miR-200 and miR-183 families in the GCP-miR network (Fig. 7). The identified module consisted of seven miRNAs, two lncRNAs and five mRNAs. All targets were significantly up-regulated in LSGC, and their expression levels were significantly negatively correlated with the miRNA expression in TCGA datasets and the validation cohort. The combination of miRNAs yielded a highly significant predictive power for patient survival. The model constructed from the six genes in the module could stratify the ESGC and LSGC in independent GC samples. Finally, the contribution of the module to the invasion and migration of GC cells was validated in *vitro*. Therefore, the miR-200/183 family-mediated module can be potential clinical biomarker for GC.

However, there are still many challenges and validation is required for their clinical application. This study has a few limitations. First, the validation cohort was relatively small, which might result in potential performance bias of the model. Second, the follow-up information of the patients in the validation cohort was not sufficient to study the overall survival and to evaluate the prognostic role of the module biomarker. Third, further research is also warranted on the functions of the module in *vivo*. Large-scale prospective studies are needed to validate the prognosis value of miR-200/183 family-mediated module. In future, we will make efforts to perform large and confirmatory prospective studies to consolidate the findings in present study.

## Conclusions

Identifying functional modules in the cancer progression is a challenging task. Our AI-assisted bioinformatics model based on multimodal data revealed a highly modular architecture and indicated that seven miRNAs from the miR-200 and miR-183 families were key regulators in GC progression. The candidate module may serve as an indicator of GC progression and a potential marker to stratify patients with ESGC versus LSGC. Our findings suggest that this module is a “pluripotent module” in gene regulatory network as the two sides of a coin, providing a roadmap to investigate new diagnostic and therapeutic opportunities.

## Supplementary Information

**Additional file 1: Figure S1.** Expression of RNAs with high RNs scores in GC cell lines. **Figure S2.** Correlation plot of expression of genes in the module in TCGA dataset. Each cell contains the corresponding correlation coefficient and p-value, and its color indicates correlation according to the color key. **Figure S3.** Kaplan–Meier survival curve of patients in high-risk and low-risk groups in test cohort and whole cohort of TCGA. **Figure S4.** Correlation plot of expression of genes in the module in our newly collected clinical samples. Each cell contains the corresponding correlation coefficient and p-value, and its color indicates correlation according to the color key. **Table S1.** Demographic information for patients with GC in TCGA dataset and validation cohort. **Table S2.** Primer sequences used for qRT-PCR. **Table S3.** Sequences of miRNA mimics. **Table S4.** Parameters for cox regression model.

## Data Availability

The data and materials in this study are available from the corresponding author on request.

## Abbreviations

AUC: Area under the curve
DEGs: Differential expressed genes
ESGC: Early stage gastric cancer
GC: Gastric cancer
GO: Gene ontology
GCP-miR network: GC progression-specific miRNA mediated network
LSGC: Late stage gastric cancer
lncRNAs: Long non-coding RNAs
miRNAs: MicroRNAs
NT: Non-tumorous adjacent
*S**CC*: Spearman Correlation Coefficient
SVM-RFE: Support Vector Machine methods based on a Recursive Feature Elimination
TCGA: The Cancer Genome Atlas
