# Supplementary material for: MiR-200/183 family-mediated module biomarker for gastric cancer progression: an AI-assisted bioinformatics method with experimental functional survey
Source: J Transl Med. 2023 Mar 2;21:163. doi: 10.1186/s12967-023-04010-z (PMC9983275; doi:10.1186/s12967-023-04010-z)
Supplement: Supplementary file 1 — Additional file 1: Figure S1. Expression of RNAs with high RNs scores in GC cell lines. Figure S2. Correlation plot of expression of genes in the module in TCGA dataset. Each cell contains the corresponding correlation coefficient and p-value, and its color indicates correlation according to the color key. Figure S3. Kaplan–Meier survival curve of patients in high-risk and low-risk groups in test cohort and whole cohort of TCGA. Figure S4. Correlation plot of expression of genes in the module in our newly collected clinical samples. Each cell contains the corresponding correlation coefficient and p-value, and its color indicates correlation according to the color key. Table S1. Demographic information for patients with GC in TCGA dataset and validation cohort. Table S2. Primer sequences used for qRT-PCR. Table S3. Sequences of miRNA mimics. Table S4. Parameters for cox regression model. [file 12967_2023_4010_MOESM1_ESM.docx]

# Additional Materials


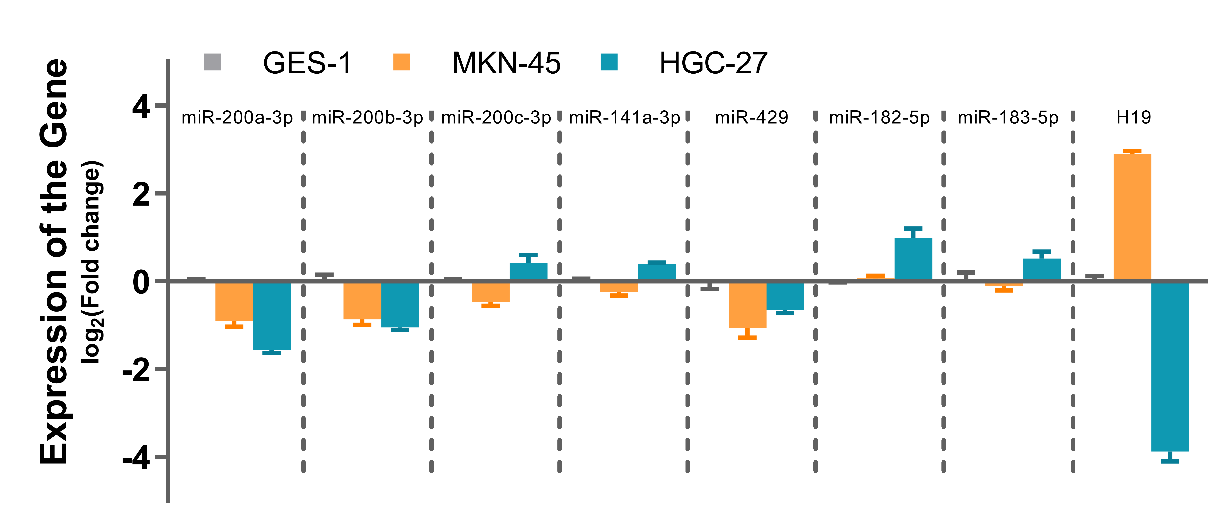


**Figure S1.** The expression of RNAs with high *RNs* scores in GC cell lines.

**
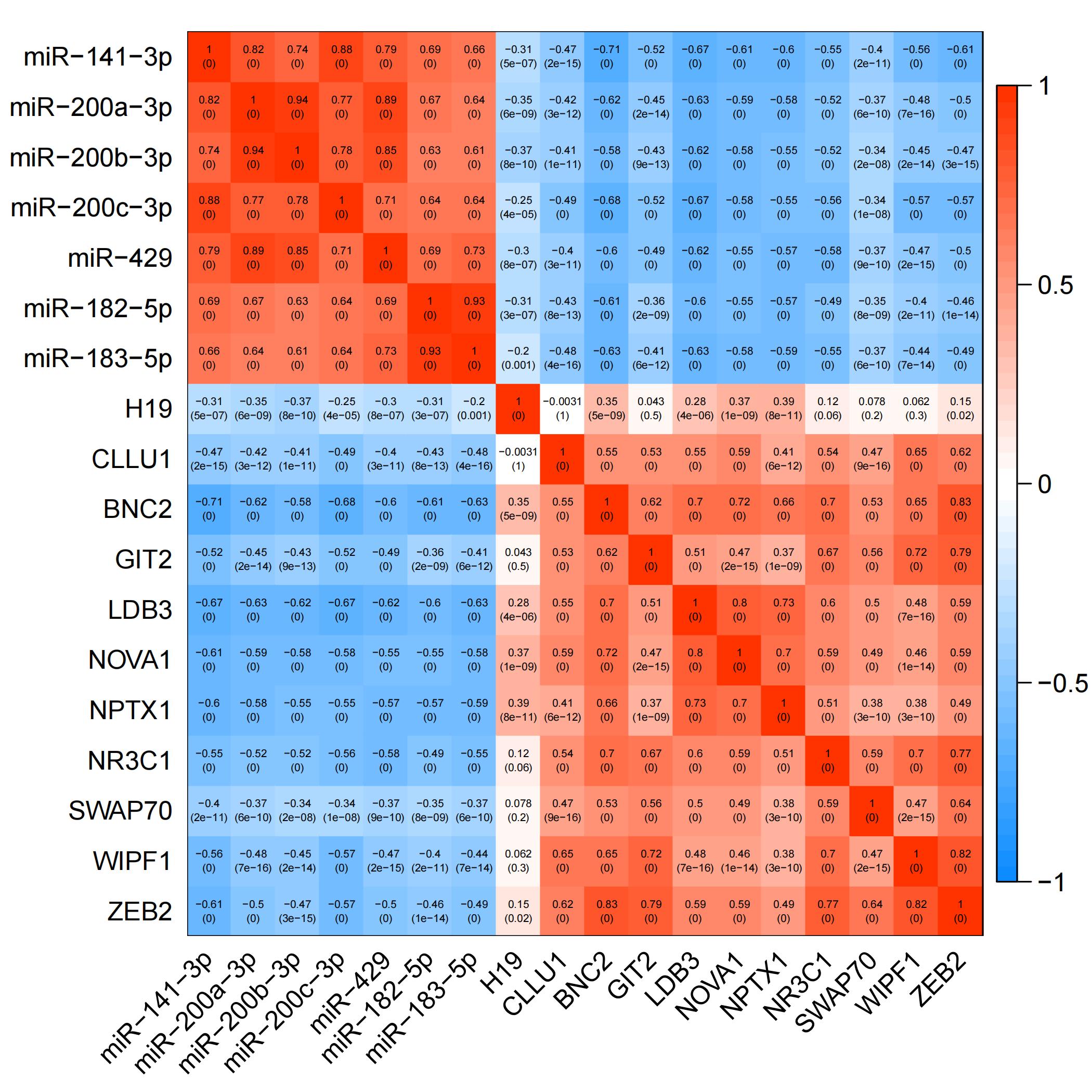
**

**Figure. S2** Correlation plot of expression of genes in the module in TCGA dataset. Each cell contains the corresponding correlation coefficient and *p-value*, and its color indicates correlation according to the color key.

**
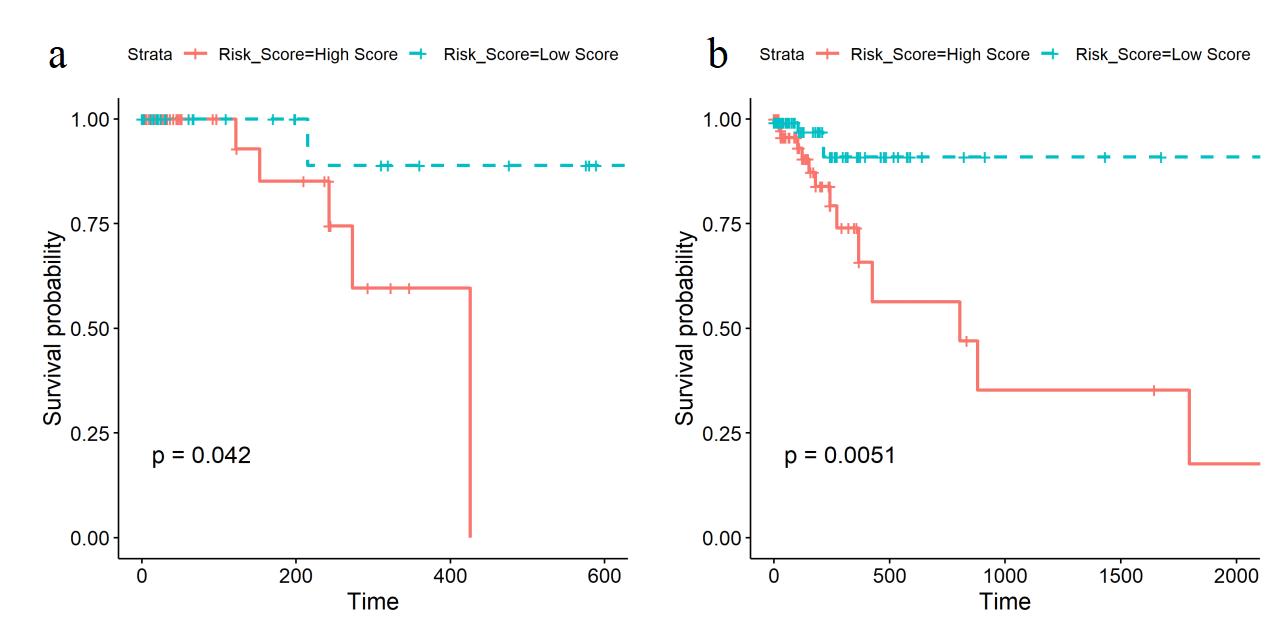
**

**Figure. S3** Kaplan-Meier survival curve of patients in high-risk and low-risk groups in (a) test cohort and (b) whole cohort of TCGA.


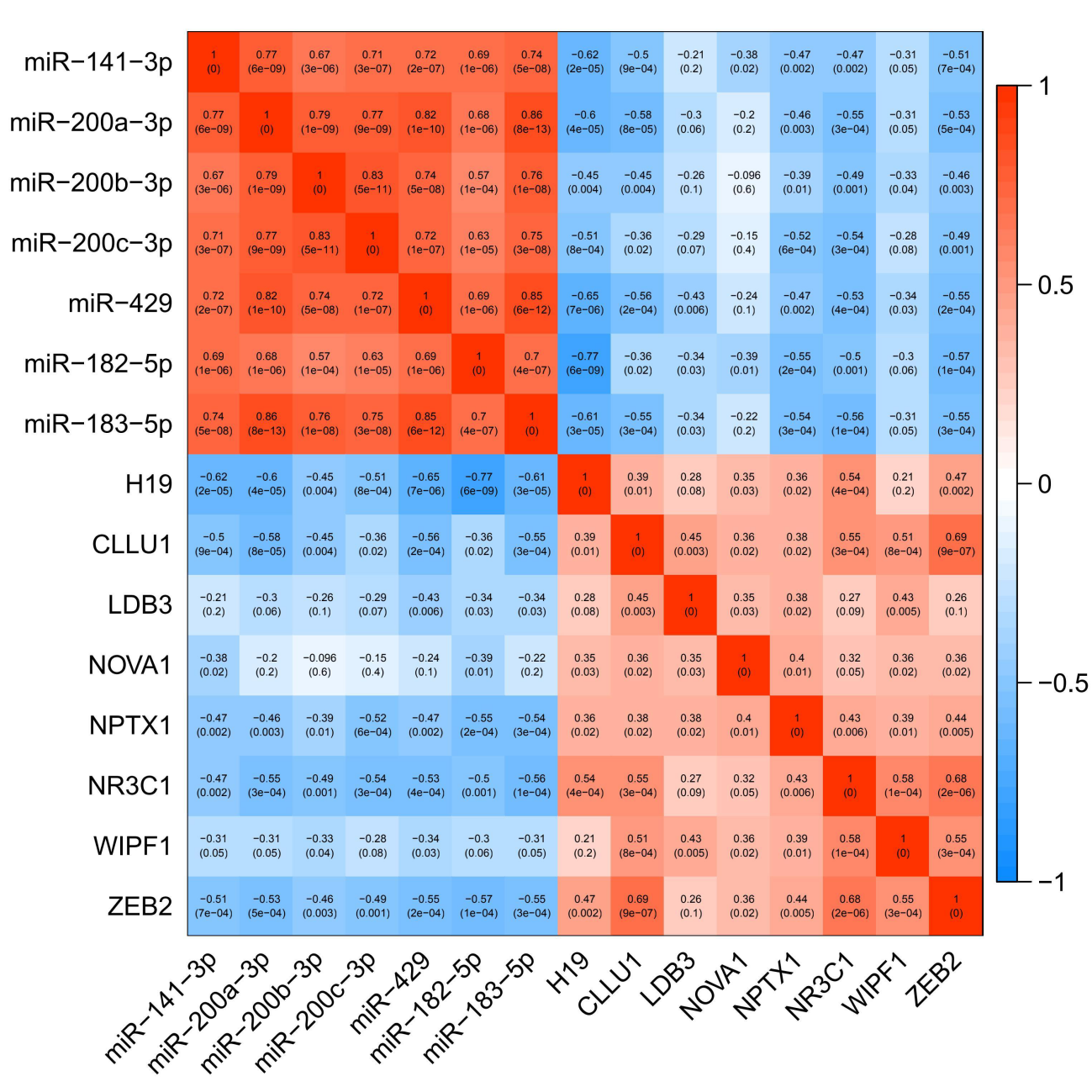
 **Figure. S4** Correlation plot of expression of genes in the module in our newly collected clinical samples. Each cell contains the corresponding correlation coefficient and *p-value* and its color indicates correlation according to the color key.

Table S1. Demographic information for patients with GC in TCGA dataset and validation cohort.

| **Clinical information** | **TCGA** | **Validation Cohort** |
| --- | --- | --- |
| **Gender** |  |  |
| Male | 158 | 17 |
| Female | 99 | 3 |
| **Years** | 34~90 | 45~85 |
| **Pathological TNM stage** |  |  |
| Stage I | 35 | 2 |
| Stage II | 94 | 7 |
| Stage III | 103 | 11 |
| Stage IV | 25 | 0 |
| **Primary tumor invasion depth** |  |  |
| T_1-2_ | 74 | 5 |
| T_3-4_ | 183 | 15 |
| **Lymph node metastasis** |  |  |
| GC without lymph node metastasis ( N_0_ ) | 87 | 7 |
| GC with lymph node metastasis ( N_1-3_) | 169 | 13 |
| Cannot be assessed (N_x_) | 1 | 0 |
| **Distant metastasis** |  |  |
| GC without distant metastasis ( M_0_ ) | 230 | 20 |
| GC with distant metastasis ( M_1_ ) | 16 | 0 |
| Cannot be assessed (M_x_) | 11 | 0 |
| **Median survival time (day)** | 29 | 1626 |
| **Vital state** |  |  |
| Alive | 238 | 9 |
| Dead | 19 | 5 |
| Unavailable | 0 | 6 |

TNM: Tumor Node Metastasis

**Table S2** Primer sequences used for qRT-PCR

| **Primers** | **Sequences (5’-3’)** |
| --- | --- |
| hsa-miR-141-3p-F | ACACTCCAGCTGGGTAACACTGTCTGGTAA |
| hsa-miR-141-3p-R | CTCAACTGGTGTCGTGGAGTCGGCAATTCAGTTGAGCCATCTTT |
| hsa-miR-200a-3p-F | ACACTCCAGCTGGGTAACACTGTCTGGTAA |
| hsa-miR-200a-3p-R | CTCAACTGGTGTCGTGGAGTCGGCAATTCAGTTGAGACATCGTT |
| hsa-miR-200b-3p-F | ACACTCCAGCTGGGTAATACTGCCTGGTAA |
| hsa-miR-200b-3p-R | CTCAACTGGTGTCGTGGAGTCGGCAATTCAGTTGAGTCATCATT |
| hsa-miR-200c-3p-F | ACACTCCAGCTGGGTAATACTGCCGGGTAAT |
| hsa-miR-200c-3p-R | CTCAACTGGTGTCGTGGAGTCGGCAATTCAGTTGAGTCCATCAT |
| hsa-miR-429-F | ACACTCCAGCTGGGTAATACTGTCTGGTAA |
| hsa-miR-429-R | CTCAACTGGTGTCGTGGAGTCGGCAATTCAGTTGAGACGGTTTT |
| hsa-miR-182-5p-F | ACACTCCAGCTGGGTTTGGCAATGGTAGAACT |
| hsa-miR-182-5p-R | CTCAACTGGTGTCGTGGAGTCGGCAATTCAGTTGAGAGTGTGAG |
| hsa-miR-183-5p-F | ACACTCCAGCTGGGTATGGCACTGGTAGAA |
| hsa-miR-183-5p-R | CTCAACTGGTGTCGTGGAGTCGGCAATTCAGTTGAGAGTGAATT |
| U6-F | CTCGCTTCGGCAGCACA |
| U6-R | AACGCTTCACGAATTTGCGT |
| NR3C1-F | ACAGCATCCCTTTCTCAACAG |
| NR3C1-R | AGATCCTTGGCACCTATTCCAAT |
| LDB3-F | CTATCTCCCGGATCACACCAG |
| LDB3-R | GTGAGGCTCAAGTTGTAGCTG |
| NOVA1-F | GGGTTCCCATAGACCTGGAC |
| NOVA1-R | CGCTCAGTAGTACCTGGGTAA |
| NPTX1-F | CACCGAGGAGAGGGTCAAGAT |
| NPTX1-R | CAGGGCGGTTGTCTTTCTGA |
| ZEB2-F | CAAGAGGCGCAAACAAGCC |
| ZEB2-R | GGTTGGCAATACCGTCATCC |
| CLLU1-F | CTTGGACTGAATTACACTGCC |
| CLLU1-R | CCACTTTTAGAGGCTTCATTT |
| H19-F | ATCGGTGCCTCAGCGTTCGG |
| H19-R | CTGTCCTCGCCGTCACACCG |
| β-actin-F | AAGGAGCCCCACGAGAAAAAT |
| β-actin-R | ACCGAACTTGCATTGATTCCAG |

**Table S3** Sequences of miRNA mimics

| miRNA mimics | Sequence |
| --- | --- |
| hsa-miR-183-5p mimics | S: UAUGGCACUGGUAGAAUUCACU  AS: UGAAUUCUACCAGUGCCAUAUU |
| hsa-miR-429 mimics | S: UAAUACUGUCUGGUAAAACCGU  AS: GGUUUUACCAGACAGUAUUAUU |

**Table S4.** Parameters for cox regression model.

| **miRNA** | **HR** | **95% CI** |
| --- | --- | --- |
| miR-200b-3p | 1.032 | 0.138-7.748 |
| miR-141-3p | 1.074 | 0.237-4.862 |
| miR-200c-3p | 0.981 | 0.191-5.035 |
| miR-200a-3p | 0.895 | 0.145-5.524 |
| miR-429 | 1.165 | 0.328-4.136 |
| miR-182-5p | 0.577 | 0.117-2.849 |
| miR-183-5p | 1.387 | 0.306-6.284 |
